# Integration of an LPAR1 Antagonist into Liposomes Enhances Their Internalization and Tumor Accumulation in an Animal Model of Human Metastatic Breast Cancer

**DOI:** 10.1021/acs.molpharmaceut.3c00348

**Published:** 2023-10-16

**Authors:** Rudolf G. Abdelmessih, Jiaming Xu, Francisco R. Hung, Debra T. Auguste

**Affiliations:** Department of Chemical Engineering, Northeastern University, 360 Huntington Avenue, Boston, Massachusetts 02115, United States

**Keywords:** LPAR1, LPAR1 antagonist, Ki161425, drug delivery, molecular dynamics simulation, metastatic breast cancer

## Abstract

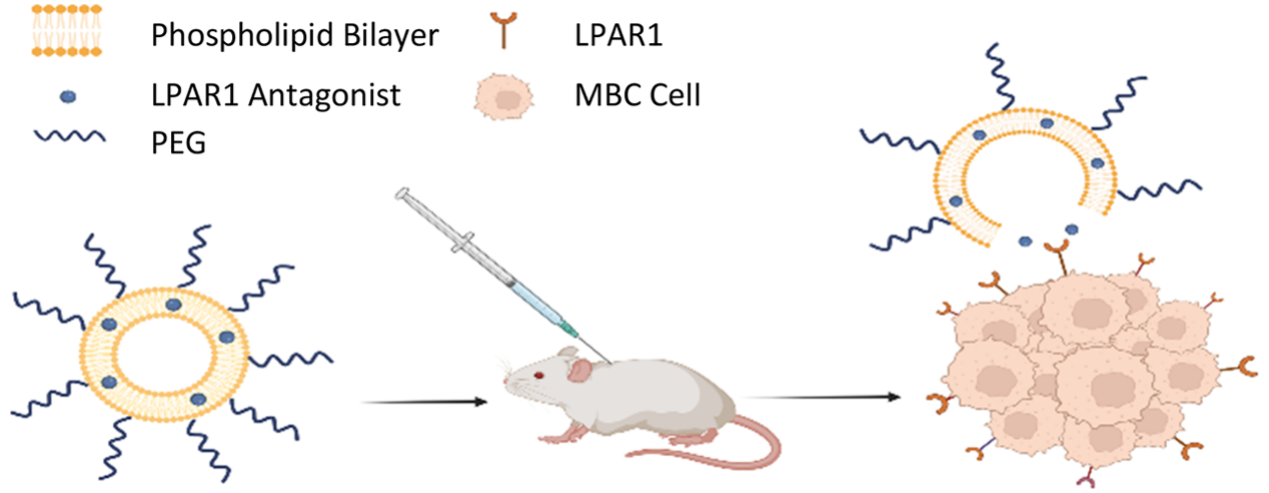

Lysophosphatidic acid receptor 1 (LPAR1) is elevated in breast cancer. The deregulation of LPAR1, including the function and level of expression, is linked to cancer initiation, progression, and metastasis. LPAR1 antagonists, AM095 or Ki16425, may be effective therapeutic molecules, yet their limited water solubility hinders in vivo delivery. In this study, we report on the synthesis of two liposomal formulations incorporating AM095 or Ki16425, embedded within the lipid bilayer, as targeted nanocarriers for metastatic breast cancer (MBC). The data show that the Ki16425 liposomal formulation exhibited a 50% increase in internalization by MBC mouse epithelial cells (4T1) and a 100% increase in tumor accumulation in a mouse model of MBC compared with that of a blank liposomal formulation (control). At the same time, normal mouse epithelial cells (EpH-4Ev) internalized the Ki16425 liposomal formulation 25% lesser than the control formulation. Molecular dynamics simulations show that the integration of AM095 or Ki16425 modified the physical and mechanical properties of the lipid bilayer, making it more flexible in these liposomal formulations compared with liposomes without drug. The incorporation of an LPAR1 antagonist within a liposomal drug delivery system represents a viable therapeutic approach for targeting the LPA–LPAR1 axis, which may hinder the progression of MBC.

## Introduction

Metastatic breast cancer (MBC) is a late-stage form of breast cancer.^1^ Approximately 30% of earlier-stage breast cancers recur in a more advanced or metastatic form,^2,3^ in which cases the disease prognosis is poor.^3^ Standard therapeutic protocols for the treatment of MBC rely mainly on chemotherapeutics that are administered systemically.^4^ The detrimental side effects of these drugs, caused mainly by off-target toxicities, limit their overall therapeutic benefits.^4^ Tumor-targeted drug nanocarriers can (1) reduce the undesirable side effects of the chemotherapeutics,^5^ (2) increase the bioavailability of anticancer drugs,^5^ (3) mitigate drug resistance,^5−7^ and (4) improve the overall therapeutic outcomes and survival of cancer patients.^5^

Lysophosphatidic acid receptors (LPARs) are a family of G-protein-coupled receptors that are activated by LPA—a native bioactive phospholipid—and play an important role in the proliferation, survival, and migration of cancer cells.^8−11^ The dysregulation of the LPAR levels and function is linked to cancer initiation, progression,^10,12−14^ and metastasis.^11,14−16^ Numerous studies demonstrated that LPAR1^11,17−19^ and LPAR3^19,20^ are overexpressed in breast cancer, including triple-negative breast cancer. Moreover, the aberrant expression of LPAR1 was reported in primary cancer cells across multiple organs,^21^ including ovaries,^22^ liver,^23,24^ stomach,^25^ pancreas,^26,27^ lungs,^24,28^ brain (glioblastoma),^29−31^ and bones.^32^ Recent studies reported that silencing^18^ or knocking out LPAR1^33^ inhibited cancer metastasis. Given these data, the LPA–LPAR axis has emerged as a viable target in the treatment of MBC.^34^

Ki16425^10,35^ and AM095^36,37^ are selective LPAR antagonists that can suppress cellular functions mediated by specific LPA receptors. Both Ki16425 and AM095 are composed of a polar carboxyl group and a largely hydrophobic part; this structure enables them to selectively interact with the binding site of LPAR1, which consists of an extracellular polar recognition region and a transmembrane hydrophobic core, as evident from the crystal structure of the LPAR1 receptor bound to similarly structured agonist and antagonist molecules.^38,39^

Ki16425 was shown to selectively inhibit the short-term cellular responses mediated by LPAR1 and LPAR3 activated by treatment with LPA, such as Ca^2+^ mobilization, inositol phosphate response, and adenosine cyclic 3′,5′-phosphate (cAMP) accumulation, but had a small effect on the LPAR2-mediated responses.^40^ Ki16425 also inhibited long-term cellular responses such as DNA synthesis and cell migration induced by treatment with LPA in 3T3 fibroblasts.^40^ The inhibition constant, *K*_i_, value for Ki16425 was estimated from the inositol phosphate responses in RH7777 cells transfected with LPAR1 to be 0.34 μM.^40^ A similar *K*_i_ value of 0.25 μM was estimated from a GTP_γ_S binding assay in HEK293T cells transfected with cDNAs encoding LPAR1.^40^ The small difference in the *K*_i_ values between the different assays was attributed to the differences in assay conditions such as temperature, reaction time, and cell type.^40^ Treatment with Ki16425 reduced cell proliferation and inhibited cell migration in glioblastoma, an aggressive primary brain tumor.^29^

AM095 selectively binds and inhibits the cellular processes mediated by LPAR1.^36,37^ AM095 was shown to inhibit the LPA-induced calcium flux in Chinese hamster ovary (CHO) cells transfected with human or mouse LPAR1.^37^ The half-maximal inhibitory concentration (IC_50_) for AM095 was estimated using a GTP_γ_S binding assay in CHO cells overexpressing human or mouse LPAR1 to be 0.98 and 0.73 μM, respectively.^36^ AM095 was also shown to inhibit the chemotaxis modulated by LPAR1 in human A2058 melanoma cells and mouse CHO cells transfected with LPAR1.^36^ Treatment with AM095 suppressed the progression of hepatocellular carcinoma in vivo.^41^ In addition, the treatment of ex vivo human liver tissues with AM095 for 48 h suppressed the high-risk genes associated with hepatocellular carcinoma as well as restored the low-risk genes associated with the disease.^41^

Ki16425 and AM095 are effective anticancer agents; yet, the tumor delivery of these water-insoluble compounds remains a challenge to achieve therapeutic results.

We have previously demonstrated the benefits to utilizing the LPA–LPAR1 axis for targeting MBC in an animal tumor model.^42,43^ Given the structure of the binding site of LPAR1,^38,39^ selective LPAR1 antagonists are mostly water-insoluble; therefore, lipid nanoparticles (e.g., liposomes) are an ideal platform for delivery since these drugs can be embedded into the lipid bilayer. Nevertheless, the integration of the LPAR1 antagonists into the lipid bilayer is expected to alter the lipid packing and lipid molecule dynamics within the membrane as well as the overall mechanical properties of the lipid membrane. The objective of this study was to synthesize liposomes incorporating LPAR1 antagonists (L-aLPAR1) and to evaluate their ability to increase in vitro internalization and in vivo targeting in an animal model of MBC. Furthermore, using molecular dynamics (MD) simulations, we investigated how the structural changes in the lipid bilayer may affect the mechanical properties of the liposomes and in turn the way in which they interact with the cancer cells. Our data support the premise that L-aLPAR1 liposomes constitute a viable drug delivery platform that can be leveraged for targeting the LPA–LPAR1 axis in the treatment of MBC.

## Materials and Methods

### Chemicals

DOPC: 1,2-dioleoyl-*sn*-glycero-3-phosphocholine, LPA: 1-oleoyl-2-hydroxy-*sn*-glycero-3-phosphate (sodium salt), and DSPE-PEG (2 kDa) carboxylic acid: 1,2-distearoyl-*sn*-glycero-3-phosphoethanolamine-*N*-[carboxy(polyethylene glycol)-2000] (sodium salt) were purchased from Avanti Polar Lipids (Alabaster, AL). Ethanol 200 proof, ACS reagent, ≥99.5% was purchased from Thermo Fisher Scientific (Waltham, MA). AM095: sodium; 2-[4-[4-[3-methyl-4-[[(1*R*)-1-phenylethoxy]carbonylamino]-1,2-oxazol-5-yl]phenyl]phenyl]acetate and Ki16425: 3-[[[4-[4-[[[1-(2-chlorophenyl)ethoxy]carbonyl]amino]-3-methyl-5-isoxazolyl]phenyl]methyl]thio]-propanoic acid were purchased from MedChemExpress LLC (Monmouth Junction, NJ). DiR: 1,1′-dioctadecyl-3,3,3′,3′-tetramethylindotricarbocyanine iodide and DiO: benzoxazolium, 3-octadecyl-2-[3-(3-octadecyl-2(3*H*)-benzoxazolylidene)-1-propenyl]-, perchlorate were purchased from Biotium (Fremont, CA). Dimethyl sulfoxide (DMSO) was purchased from MilliporeSigma (Burlington, MA). Quantum Simply Cellular anti-Mouse IgG microspheres were purchased from Bangs Laboratories, Inc. (Fishers, IN). EDG-2 antibody (B-10) phycoerythrin (PE) and EDG-7 antibody (C-7) were purchased from Santa Cruz Biotechnology, Inc. (Dallas, TX). Tumor necrosis factor alpha (TNF-α) was purchased from R&D Systems, Inc. (Minneapolis, MN).

### Cell Lines and Cell Culture

Mouse normal epithelial cells (EpH4-Ev), mouse tumorigenic epithelial cells from the mammary gland tissue (4T1), human normal epithelial cells (MCF 10A), human tumorigenic epithelial cells from the mammary gland tissue (MDA-MB-231), Dulbecco’s modified Eagle’s medium (DMEM), Roswell Park Memorial Institute (RPMI)-1640 medium, fetal bovine serum (FBS), and calf bovine serum (CBS) were purchased from American Type Cell Culture (Manassas, Virginia). Puromycin and mammary epithelial cell growth medium (PromoCell) were purchased from Thermo Fisher Scientific (Waltham, MA). The EpH4-Ev cells were cultured using DMEM supplemented with 10% CBS and 1.2 μg/mL puromycin. The 4T1 cells were cultured using RPMI-1640 medium supplemented with 10% FBS. MCF 10A cells were cultured using mammary epithelial cell growth medium (PromoCell). The MDA-MB-231 cells were cultured using DMEM supplemented with 10% FBS. All the cell lines were incubated at 37 °C in 95% air, 5% CO_2_, humidified atmosphere.

### Synthesis and Characterization of the Liposomal Formulations

Four liposomal formulations were prepared using the solvent injection method.^44^ Briefly, DOPC, 5 mol % DSPE-PEG (2 kDa) carboxylic acid, and 1 mol % DiIC_18_(7) (DiR) were dissolved in ethanol and stirred—alone, or with 20 mol % of either LPA, AM095, or Ki16425—at 25 °C for 45 min and then slowly injected into phosphate-buffered saline (PBS), pH 7.4, while constantly stirring, to formulate unilamellar liposomal vesicles. The formulated liposomes were then centrifuged at 3000*g* in a 100 kDa molecular-weight-cutoff centrifugal filter tube for 1 h and resuspended in PBS. This step was repeated three times to remove all organic solvents, and the samples were concentrated down to 2.69 mM. The first filtrate was preserved for spectrophotometric analysis.

The amount of the free nonentrapped drug was determined by measuring the absorbance of two sets of three samples of the first filtrate of the liposomal formulations of AM095 and Ki16425 at λ = 330 and λ = 295 nm, respectively, using a microplate reader (SpectraMax Gemini-XPS, Molecular Devices, Sunnyvale, CA) and comparing the results to standard curves of absorbance vs concentration of AM095 or Ki16425.

The encapsulation efficiency (EE) values of AM095 and Ki16425 were determined as follows
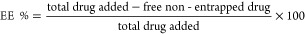
1

To measure the drug release (DR) of AM095 and Ki16425 from their respective liposomal formulations, the liposomes were dialyzed against PBS (pH 7.4) that contained 0.1% (w/v) Tween 20 for 24 h using a 10 kDa molecular-weight-cutoff dialysis cassette.^43^ Afterward, the AM095 and Ki16425 liposomes were retrieved, and their absorbance was measured at λ = 345 and 320 nm, respectively; and the results were compared to standard curves of absorbance vs concentration. DR was determined as follows
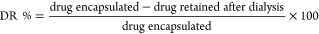
2

The liposomal formulations were labeled with DiIC_18_(7) (DiR)—a near-infrared fluorescent dye, λ_Ex_/λ_Em_ = 748/780 nm^45^—to enable the tracking of liposomes using flow cytometry in the in vitro studies and in vivo imaging system (IVIS) in the animal studies. For the purpose of characterization of the nanoparticles, similar formulations were synthesized with 1% DiOC_18_(3) (DiO) instead of DiR, as DiO does not interfere with dynamic light scattering (DLS) measurements, unlike DiR. After synthesis, the formulations were stored at 4 °C.

DiIC_18_(7) is a type of carbocyanine (polymethine) dye.^45^ The amphiphilic structure of the dialkylcarbocyanine dyes (e.g., DiI, DiO, DiD, and DiR) resembles the general structure of biological lipids, which enables the labeling of the phospholipid bilayer membranes, lipoproteins, and other lipid-based molecular structures for a variety of applications such as cell tracing and in vivo imaging.^45−75^ These fluorophores intercalate within the phospholipid membranes through noncovalent intermolecular interactions such that the charged part of the fluorophore resides on the polar surface of the membrane, while the lipophilic alkyl tails embed into the hydrophobic part.^46^ These lipophilic carbocyanine dyes generally do not transfer from labeled to unlabeled membranes but diffuse within the membrane.^46,76^

To confirm the stability of the labeled formulations under physiological conditions, the release of the fluorescent probe was measured. The liposomes were incubated in PBS (pH 7.4) that contained 10% FBS at 37 °C for 24 h,^67^ and then, a sample of each formulation was centrifuged at 3000*g* in a 10 kDa molecular-weight-cutoff centrifugal filter tube for 1 h. Afterward, the filtrates were collected, and their fluorescence was measured (λ_Ex_/λ_Em_ = 748/780 nm) using a microplate reader (SpectraMax Gemini-XPS, Molecular Devices, Sunnyvale, CA). No fluorescent signal was detected in the filtrate of any of the formulations.

Effective diameter, polydispersity, and diffusion coefficient were measured by DLS, using a particle size analyzer (Brookhaven Instruments Corporation, Holtsville, NY), and the data were collected using BIC particle sizing software. ζ-potential, mobility, and conductance were measured in diH_2_O at 25 °C using BIC phase analysis light scattering (Brookhaven Instruments Corporation).

### Quantitative Analysis and Comparison of Cellular Antigen Expression of LPAR1 and LPAR3 in MBC Cells and Normal Epithelial Cells

The levels of expression of LPAR1 and LPAR3 were measured in mouse MBC cells (4T1) and normal mouse epithelial cells (EpH-4Ev). Both cell lines were treated with the same live-staining protocol as follows. After a monolayer of cells reached 80% confluency in a culture flask, the medium was vacuumed off, and the cells were rinsed with 1× PBS, which was then vacuumed off. The cells were then treated with 0.2% ethylenediaminetetraacetic acid (EDTA) in PBS and incubated at 37 °C in humidified air with 5% CO_2_ for 5–10 min. After the cells detached, 5 mL of full medium was added to neutralize the EDTA. The cells were counted using a hemacytometer, centrifuged and resuspended in 1X PBS to a final concentration of 1 × 10^6^ cells/mL. Nonspecific receptor binding was blocked by incubating the cells with normal mouse IgG at the concentration of 1 μg IgG/1 × 10^6^ cells for 10 min. Subsequently, 1 μg of PE-conjugated antibodies specific to either LPAR1 or LPAR3 was added to microcentrifuge tubes, to which 100 μL of cell suspension was added. The tubes were then vortexed and incubated for 15–30 min on ice in the dark. Next, excess antibodies were washed off by adding 1.5–2 mL of PBS to each tube, centrifuging and resuspending in 500 μL of 1% paraformaldehyde. Afterward, the mean fluorescence intensity was measured for all the samples using a CytoFLEX flow cytometer (Beckman Coulter). In another set of samples, the 4T1 cells were treated with 21.8 μM LPA for 96 h prior to staining with LPAR1 and LPAR3 antibodies following the same protocol.

Data obtained from flow cytometry along with Quantum Simply Cellular anti-Mouse IgG microspheres were used to determine the antibody binding capacity of cells for LPAR1 and LPAR3 according to the accompanying protocol.

### Measurement of Cellular Internalization of L-aLPAR1 In Vitro

The EpH-4Ev and 4T1 cells were seeded in two 24-well plates each (1 × 10^5^ cells in 500 μL whole medium/well) and incubated at 37 °C in a 5% CO_2_ humidified air overnight to allow the cells to attach to the wells’ surface. The next day, the media were removed, and the wells were rinsed with 1X PBS. Next, 0.538 mM of each liposomal formulation in a total of 500 μL of whole medium was added to the wells in triplicates on each plate and cells incubated at 37 °C in 5% CO_2_ humidified air—one plate (for each cell line) was incubated for 4.5 h and another for 7.5 h. Afterward, the media were removed, and the cells were rinsed with 1X PBS and then treated with 200 μL of trypsin per well until detachment, and then 600 μL of new medium was added to each well to neutralize trypsin. Subsequently, the cells were transferred to microcentrifuge tubes, and the levels of internalization were determined by measuring the mean fluorescence intensity using the CytoFLEX flow cytometer. The same protocol was followed to measure the internalization of L-aLPAR1 in MCF 10A and MDA-MB-231 cells after incubation with each formulation for 3 h. In another set of samples, the 4T1 cells were treated with 100 ng/mL TNF-α and incubated at 37 °C in 5% CO_2_ humidified air overnight prior to treatment with liposomes. The same protocol was followed to determine the cellular internalization in which the cells were incubated for 3 h after the addition of liposomes.

### Determination of the Effect of LPA on the Cellular Internalization of L-aLPAR1

The 4T1 cells were seeded in a 24-well plate (5 × 10^4^ cells in 1000 μL whole medium/well) and incubated at 37 °C in a 5% CO_2_ humidified air overnight to allow the cells to attach to the wells’ surface. The next day, the media were removed, and the wells were rinsed with 1× PBS. Next, 1000 μL of whole medium was added to the cells with and without 40 μM of LPA for the control and the experimental groups, respectively, and the cells were incubated at 37 °C in a 5% CO_2_ humidified air. Twenty-four hours later, the media were removed, and the wells were rinsed with 1× PBS, and then, 0.538 mM of each liposomal formulation in a total of 1000 μL of whole medium was added to the wells in triplicates and the cells were incubated for 3 h at 37 °C in a 5% CO_2_ humidified air. Afterward, the media were removed, and the cells were rinsed with 1X PBS and then treated with trypsin until detachment, and new media were added. Subsequently, the cells were transferred to microcentrifuge tubes, and the levels of internalization were determined by measuring the mean fluorescence intensity using the CytoFLEX flow cytometer.

### Determination of the Mechanism of Cellular Internalization of L-aLPAR1

The 4T1 cells were seeded in a 24-well plate (1 × 10^5^ cells in 500 μL of whole medium/well) and incubated at 37 °C in a 5% CO_2_ humidified air overnight to allow the cells to attach to the wells’ surface. The next day, the media were removed, and the wells were rinsed with 1× PBS. Next, 500 μL of whole medium supplemented with one of the endocytosis inhibitors (at the concentration outlined in Table 1 below) was added to the wells in triplicates, and the cells were incubated at 37 °C in 5% CO_2_ humidified air for 1 h. Afterward, 0.538 mM of L-Ki16425 was added to each well, and the cells were incubated again under the same conditions for 3 h. Finally, the media were removed, and the cells were rinsed with 1× PBS and then treated with trypsin until detachment, and new media were added. The cells were then transferred to microcentrifuge tubes, and the levels of internalization were determined by measuring the mean fluorescence intensity using the CytoFLEX flow cytometer.

**Table 1:** Endocytosis Inhibitors and Their Concentrations

inhibitor	concentration (μM)
filipin	5
DNS	10
CCD	10 × 10^3^

### Animal Model and Measurement of Tumor Accumulation and Biodistribution of L-aLPAR1 In Vivo

Female immunocompetent BALB/c mice weighing 19–21 g and aged 6–8 weeks were purchased from Charles River Laboratories International, Inc. (Wilmington, MA). To construct a syngeneic model for MBC, 16 animals were randomized and divided into a control group and three experimental groups (*n* = 4). Each mouse was injected with 1.5 × 10^6^ 4T1 cells suspended in 100 μL of sterile PBS into the fourth (inguinal) mammary fat pad to create an orthotopic allograft of an MBC mass. The tumor masses were allowed to grow, their dimensions were measured, and their volumes were calculated every 2 days as follows
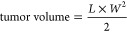
3where *L* is the length and *W* is the width of the tumor mass.

When the tumor sizes reached ∼200 mm^3^ (10 days after tumor injection), the animals were given a single injection, via retro-orbital route, of 100 μL (2.69 mM) of one of the four liposomal formulations. Accumulation of the different formulations in the tumor was measured 1.5, 3, 6, 9, 24, and 48 h after injection using IVIS Lumina II (Caliper, Hopkinton, MA). A negative control group, not injected with any liposomes, was imaged for fluorescence background. All the images were analyzed using Living Image Software (PerkinElmer). The region of interest tool was used to measure the average radiant efficiency ([p/s/cm^2^/sr]/[μW/cm^2^]) of an equal area of the animal's body in all groups where the tumor mass was constructed. Background fluorescence was subtracted from all the experimental values. At the end of the study, the animals were euthanized, and the levels of accumulation of the different liposomal formulations were measured in all organs, ex vivo, using IVIS Lumina II (Caliper, Hopkinton, MA).

### Statistical Analysis

All the experimental values were presented as the mean ± standard deviation. Statistical analysis was done using one-way analysis of variance (ANOVA) (α = 0.05, 0.01, 0.001) with post hoc Tukey test. All statistics were run on OriginPro, Version 2023. OriginLab Corporation, Northampton, MA, USA. Statistical significance was concluded when *P* ≤ α and indicated on the corresponding figures for each experiment as follows: *p < 0.05, **p < 0.01, and ***p < 0.001.

### MD Simulations

We used MD simulations to investigate the changes in the mechanical properties of the lipid bilayers of liposomes^77−81^ that the integration of AM095 and Ki16425 molecules may have effected. The DOPC lipid bilayer structure was initially assembled using the heterogeneous lipid generation tool provided by CHARMM-GUI, following established protocols.^82,83^ Each leaflet was composed of 100 DOPC lipids, with hydration provided by water layer 2.25 nm in thickness. Sodium chloride and potassium chloride ions were subsequently added to the water layer to achieve a salt concentration of 0.15 M, thus mimicking the osmolarity and pH of the buffer, where the liposomes were synthesized. Lipids and ions were modeled using the CHARMM36m all-atom force field, while water molecules were represented by the TIP3P model.^84,85^ Additionally, AM095 and Ki16425 were parameterized using the CHARMM general force field (CGenFF).^86^ To assemble the bilayer–drug complex, we employed a method in which we split the two layers of the lipid molecules by 0.3 nm, inserted a layer of 49 drug molecules of either AM095 or Ki16425—to match the 20 mol % used experimentally—between them, and then allowed the system to relax. Figure S2A,B shows snapshots of the simulations of the DOPC-AM095 and DOPC-Ki16425 systems.

The area compressibility modulus (*K*_A_)^87^ of the pure DOPC, DOPC-AM095, and DOPC-Ki16425 systems was calculated as follows
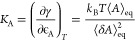
4where ϵ_A_ is the area strain defined as  and γ is the surface tension that is defined as
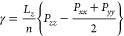
5where *P*_*xx*_, *P*_*yy*_, and *P*_*zz*_ refer to pressure in the *x*, *y*, and *z* directions.

The diffusion coefficient of the lipids and drugs (*D*)^88,89^ was calculated from the mean squared displacement (MSD) as follows
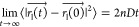
6where *n* is the dimensionality of the MSD.

Lipid acyl chain order parameter (*S*_c_)^90−92^ for acyl chain 1 and 2 of DOPC in the pure DOPC, DOPC-AM095, and DOPC-Ki16425 systems was calculated with *S*_c_ being defined for each *C*_*i*_ atom in the hydrocarbon chains as
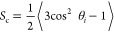
7where θ_*i*_ is the angle between the *z*-axis (the bilayer normal) of the simulation box and the bond linking carbon atoms *C*_*i*_ to *C*_*i*+1_.

Prior to conducting the MD simulations on the bilayer–drug systems, we performed several simulations to ensure the accuracy of the CGenFF force field for AM095 and Ki16425. Given the limited experimental data on the physical properties of these molecules, we determined that a comparison with their available experimental solubilities in DMSO would be the most appropriate method of validation. To this end, we modeled DMSO using the CGenFF, converted the concentration solubility of the drugs in DMSO to number solubility in 1000 molecules DMSO bulk, and then conducted simulations using 10, 15, 20, 30, and 40 molecules of each drug. The initial configuration was constructed using Packmol^93^ in a 5 × 5 × 5 nm^3^ cubic box and then relaxed by energy minimization procedure. After energy minimization, 5 nm constant-number, constant-volume, constant-temperature (NVT) ensemble and 100 ns constant-number, constant-pressure, constant-temperature (NPT) ensemble production simulations were carried out. Temperature and pressure were set at 300 K and 1 bar and controlled by the Nose–Hoover thermostat and Parrinello–Rahman barostat, respectively.^94,95^Figure S1A,B shows the radial distribution function (RDF) of the 5 systems for AM095 and Ki16425, respectively; Figure S1C,D shows the chemical structure as well as the nomenclature of all the atoms for AM095 and Ki16425, respectively. The RDF of these systems for both drugs showed an increase in density with the increase in the number of molecules from 10 up to 30; however, no significant difference was observed when the number of AM095 or Ki16425 molecules was increased beyond 30. This observation suggests that the lower limit of the saturation concentration is approximately at 20 molecules or 114 and 117 mg/mL for AM095 and Ki16425, respectively. These results are in general agreement with the available experimental data for the solubility of both drugs in DMSO, that is, 83.3 and 100 mg/mL for AM095 and Ki16425, respectively.^96,97^Figure S3A–J shows snapshots of the simulations of the different systems of AM095 and Ki16425 in DMSO, which suggest that the drug molecules tend to cluster together if the total number of AM095 or Ki16425 molecules is 15 or larger (85.5 or 87.75 mg/mL, respectively).

All MD simulations were conducted using the GROMACS (v2018.4) simulation package with graphics processing unit (GPU) accelerations.^98−101^ Seven equilibration-step simulations were performed as recommended by CHARMM-GUI, and then, a 10 ns preproduction run was conducted to assess the stability of the bilayer–drug complex. Subsequently, a 200 ns production run was carried out, employing a time step of 2 fs with pressure and temperature fixed at 1 bar and 310 K, respectively. Pressure and temperature were controlled by the Parrinello–Rahman barostat and Nose–Hoover thermostat, respectively.^94,95^ The LINCS method was used to constrain the bonds with hydrogen atoms.^102^ Particle mesh Ewald method^103^ with a real-space cutoff at 1.2 nm was used for electrostatic interaction calculations, and van der Waals interactions were cutoff at 1.0 nm and gradually switched to zero at 1.2 nm.

## Results

### 4T1 Cells Have Higher Density of LPAR1 Than Normal Epithelial Cells

**Figure 1:**
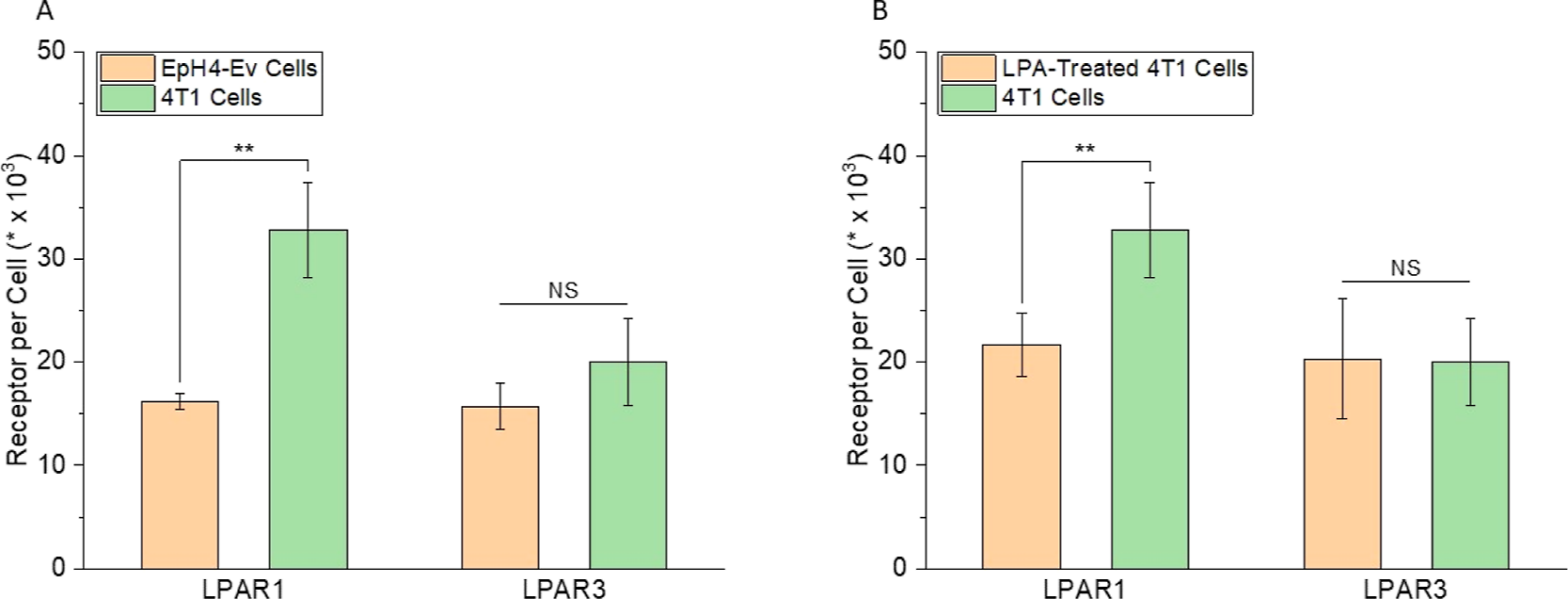
Receptor expression levels of LPAR1 and LPAR3. (A) Mouse MBC cells (4T1) showed a 100% higher density of LPAR1 compared with that of normal mouse epithelial cells (EpH4-Ev). There was no statistically significant difference in the density of LPAR3 between the two cell lines. (B) LPA-treated 4T1 cells showed 34% decrease in binding of LPAR1 antibodies compared to that in nontreated 4T1 cells. Statistical significance was determined by one-way ANOVA followed by Tukey post hoc test (*n* = 3, **p < 0.01, NS: nonsignificant).

In order to establish that the MBC model we had in this study is viable to investigate the function of an LPAR-targeted drug delivery system, we quantified the number of LPAR1 and LPAR3 receptors in mouse MBC cells, 4T1, and compared it to normal mouse epithelial cells, EpH4-Ev. Figure 1A shows that the 4T1 cells had a 100% higher density of LPAR1 compared to that of the EpH-4Ev cells. The difference in the density of LPAR3 between the two cell lines was not statistically significant.

Next, we confirmed the specificity of the antibodies used to quantify LPAR1 and LPAR3. The cells were treated with a competing ligand for the receptors (i.e., their native binding ligand, LPA). We observed a reduced level of binding of LPAR1 antibodies to the receptors as the two molecules competed for the same binding sites. Figure 1B shows that the LPA-treated 4T1 cells showed a 34% decrease in binding of LPAR1 antibodies compared to nontreated 4T1 cells. There was no significant difference in the level of binding of LPAR3 antibodies between LPA-treated 4T1 cells and nontreated 4T1 cells.

### Synthesis and Characterization of Liposomes

In this study, a total of four liposomal formulations were synthesized. The main component in all the formulations was the DOPC lipid. DSPE-PEG(2 kDa) was added to all the formulations to increase their blood circulation time^104,105^ and tumor accumulation.^106^ The two control formulations contained either no drug molecules (L-DOPC) or the native ligand LPA (L-LPA). The two experimental formulations contained one of two LPAR1 antagonists (aLPAR1): AM095 (L-AM095) or Ki16425 (L-Ki16425). The EEs of AM095 and Ki16425 were determined to be 91.9 ± 0.2 and 87.3 ± 0.3%, respectively. Over 24 h, 9.5 ± 1.0% of AM095 and 5.6 ± 0.9% of Ki16425 were released from L-AM095 and L-Ki16425, respectively. Table 2 shows the chemical structures of the molecules used to synthesize the four liposomal formulations. Table 3 describes the composition and physical properties of each formulation.

**Table 2:**
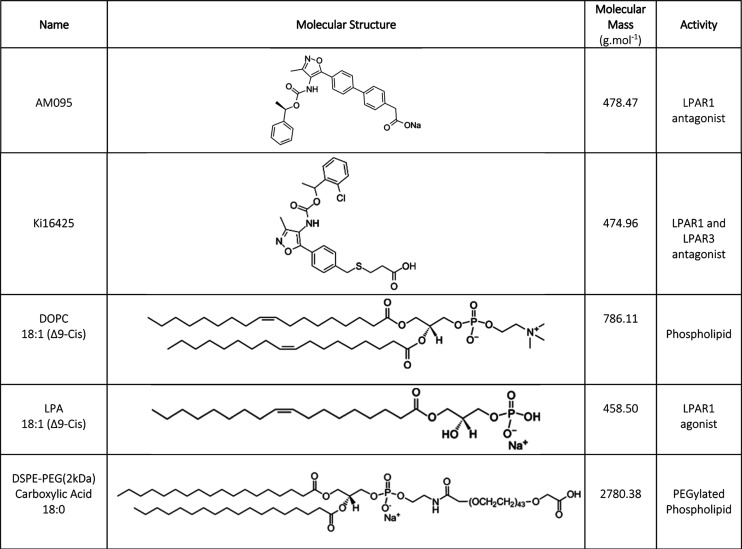
Molecules Used for the Synthesis of the Liposomal Formulations

**Table 3:** Characterization of the Liposomal Formulations Using DLS^b^

name	composition^a^	effective diameter (nm)	polydispersity index (PDI)	diffusion coefficient (cm^2^/s)	ζ-potential (mV)	mobility (μ/s)/(V/cm)	conductance (μS)
L-DOPC	DOPC (94 mol %)	53 ± 1	0.22 ± 0.01	0.92 × 10^–^^7^ ± 0.2 × 10^–^^8^	–55 ± 12	–2.9 ± 0.7	291 ± 2
L-LPA	DOPC/LPA (74:20 mol %)	41 ± 2	0.22 ± 0.02	1.19 × 10^–^^7^ ± 0.4 × 10^–^^8^	–42 ± 14	–2.2 ± 0.7	271 ± 8
L-AM095	DOPC/AM095 (74:20 mol %)	44 ± 1	0.19 ± 0.02	1.11 × 10^–^^7^ + 0.2 × 10^–^^8^	–39 ± 11	–2.1 ± 0.6	276 ± 6
L-Ki16425	DOPC/Ki16425 (74:20 mol %)	46 ± 1	0.21 ± 0.01	1.06 × 10^–^^7^ ± 0.2 × 10^–^^8^	–50 ± 3	–2.6 ± 0.2	281 ± 1

a Each composition includes 6 mol % DSPE-PEG (2 kDa).

b All the values are presented as the mean ± standard deviation. For all measurements, *n* = 3.

### Uptake of Liposome-Formulated LPAR1 Antagonists by 4T1 Cells

**Figure 2:**
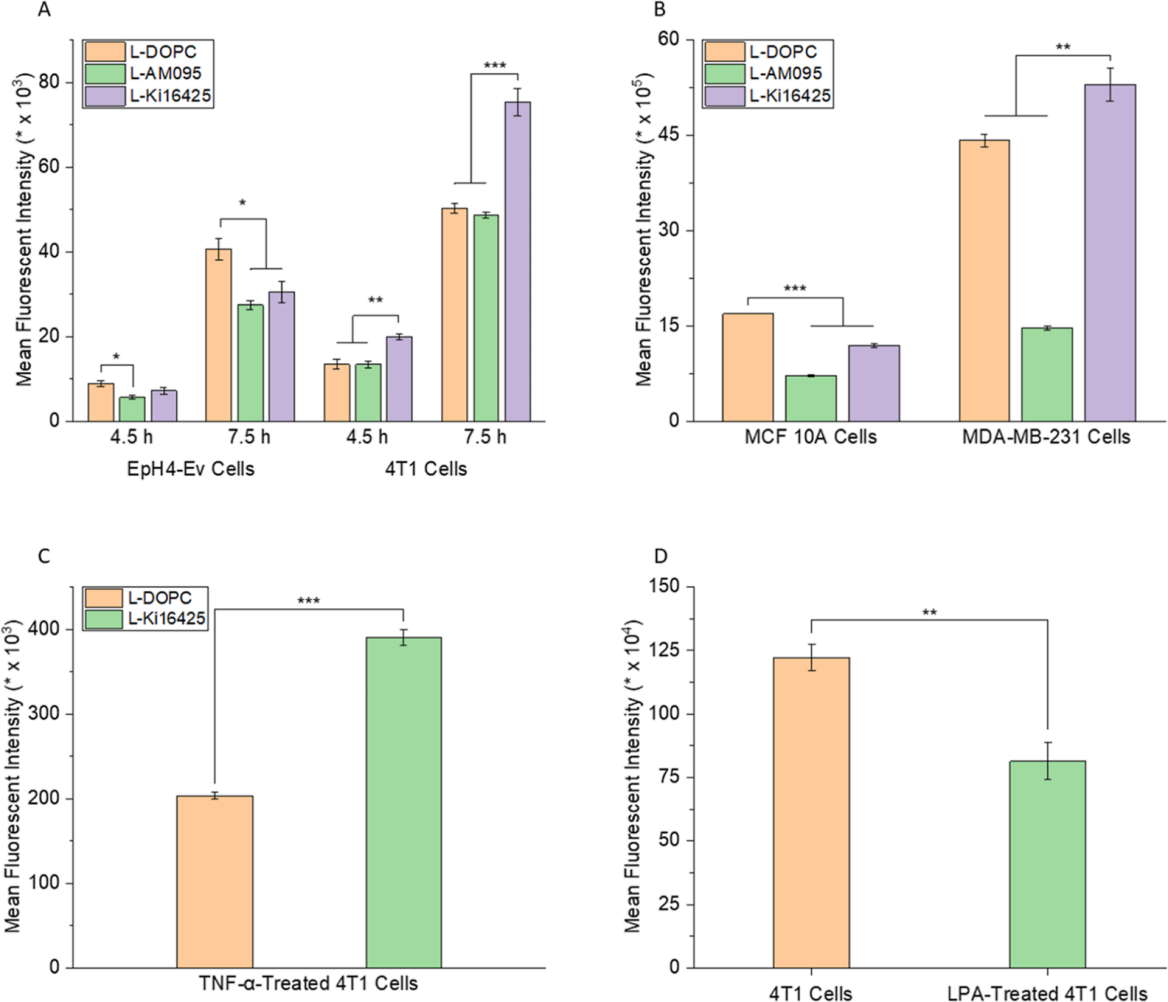
Levels of cellular internalization of L-aLPAR1 and the effect of TNF-α and LPA on cellular uptake. (A) The levels of internalization of L-aLPAR1 were measured at two time points: 4.5 and 7.5 h after incubation. At 4.5 h, the EpH4-Ev cells internalized L-DOPC 59 and 23% more than L-AM095 and L-Ki16425, respectively. Conversely, at the same time point, 4T1 cells internalized L-Ki16425 48 and 49% more than L-DOPC and L-AM095, respectively. At 7.5 h, the EpH4-Ev cells internalized L-DOPC 48 and 33% more than L-AM095 and L-Ki16425, respectively. Yet, at the same time point, the 4T1 cells internalized L-Ki16425 50% and 55% more than L-DOPC and L-AM095, respectively. (B) The internalization of L-aLPAR1 was measured in human normal and tumorigenic epithelial cells. MDA-MB-231 internalized L-Ki16425 1.2 times and 3.6 times more than L-DOPC and L-AM095, respectively, while MCF 10A cells internalized L-DOPC 2.4 times and 1.4 times more than L-AM095 and L-Ki16425, respectively. (C) L-Ki16425 was internalized by TNF-α-treated 4T1 cells 90% more than L-DOPC, after 3 h of incubation. (D) The presence of a competing ligand for LPAR1 (i.e., LPA) reduced the 4T1 cellular uptake of L-Ki16425 by 33%. Statistical significance was determined by one-way ANOVA followed by Tukey post hoc test (*n* = 3, *p < 0.05, **p < 0.01, and ***p < 0.001).

We evaluated the uptake of the L-aLPAR1 formulations relative to that of L-DOPC in tumorigenic 4T1 and normal EpH4-Ev cells. Figure 2A shows that the 4T1 cellular uptake of L-Ki16425 at 4.5 h was 48% and 49% greater than those of L-DOPC and L-AM095, respectively. At 7.5 h, the 4T1 cellular uptake of L-Ki16425 was 50% and 55% greater than that of L-DOPC and L-AM095, respectively. Conversely, in the EpH4-Ev cells, at 4.5 h, L-DOPC was internalized 59% and 23% more than L-AM095 and L-Ki16425, respectively. At 7.5 h, L-DOPC had 48% and 33% higher internalization in EpH4-Ev cells than L-AM095 and L-Ki16425, respectively.

To examine how these results translate to human cells, we tested the uptake of L-aLPAR1 in normal epithelial cells (MCF 10A) and tumorigenic epithelial cells (MDA-MB-231). Similar results were obtained as shown in Figure 2B. L-Ki16425 showed 1.2 times and 3.6 times greater internalization in MDA-MB-231 cells relative to L-DOPC and L-AM095, respectively. MCF 10A cells internalized L-DOPC 2.4 times and 1.4 times more than L-AM095 and L-Ki16425, respectively.

Mimicking an aspect of the tumor microenvironment in vitro, we evaluated L-aLPAR1 uptake by 4T1 cells in the presence of TNF-α that is implicated in inflammation-associated carcinogenesis.^107^ The 4T1 cells were treated with TNF-α prior to treatment with liposomes. Figure 2C shows that the 4T1 cellular uptake of L-Ki16425 in the presence of TNF-α was 90% more than that of L-DOPC.

Furthermore, we evaluated L-aLPAR1 uptake by 4T1 cells in the presence of a competing ligand (i.e., LPA), which is the native ligand of LPAR1. Figure 2D shows that the presence of LPA reduced the 4T1 cellular uptake of L-Ki16425 by 33%, which suggests that the uptake of L-Ki16425 is LPAR-mediated.

### Caveolin- and Clathrin-Mediated Endocytosis Control the Internalization of L-Ki16425

**Figure 3:**
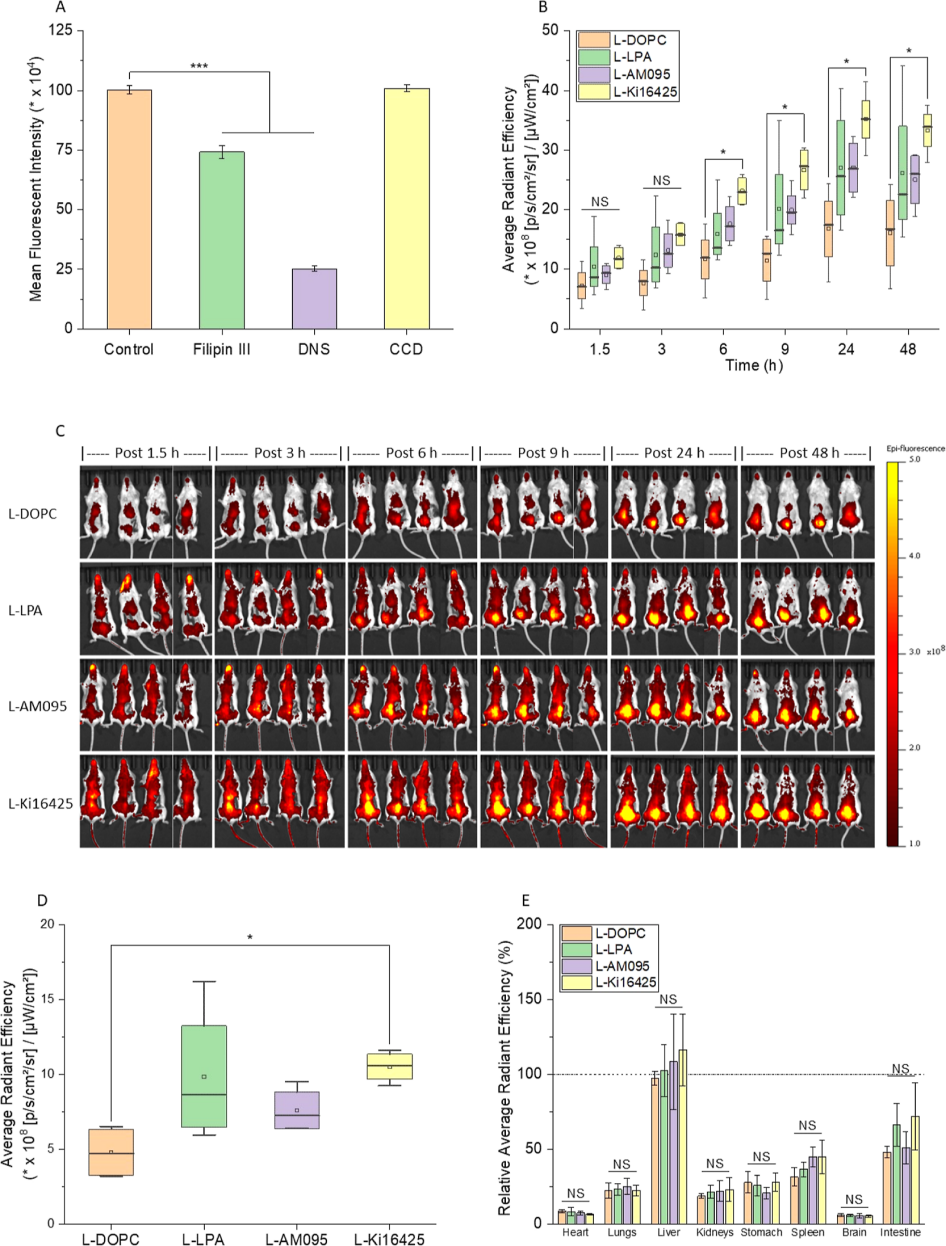
Mechanisms of cellular uptake, the level of accumulation in MBC tumor mass in live animals, and ex vivo biodistribution of L-aLPAR1. (A) 4T1 cells were treated with three different endocytosis inhibitors separately prior to treatment with L-Ki16425. No inhibitor was added to the control group prior to treatment with L-Ki16425. Filipin III and DNS reduced the internalization of L-Ki16425 by 26 and 75%, respectively, compared with the control. (B) Average radiant efficiency of liposomes in the MBC tumor regions, and (C) whole animal images across 48 h. Starting at 6 h after injection until the end of the study at 48 h after injection, L-Ki16425 had a higher level of tumor accumulation compared with the other three formulations. L-Ki16425 showed a 100%, or more, increase in accumulation over L-DOPC at 6, 9, 24, and 48 h after injection. Average radiant efficiency data in live animals were normalized by tumor volumes. (D) Level of accumulation of the different formulations of L-aLPAR1 in tumor masses ex vivo. (E) Distribution profile of L-aLPAR1 in different organs ex vivo, represented as a percentage of the level of the accumulation of liposomes in the tumor mass. The horizontal line at 100% represents the level of accumulation in the tumor mass. Ex vivo average radiant efficiency data were normalized by tumor weights. For (A), statistical significance was determined by one-way ANOVA followed by Tukey post hoc test (*n* = 3, ***p < 0.001). For (B,D, and ,E), statistical significance was determined by one-way ANOVA followed by Tukey post hoc test (*n* = 4, *p < 0.05, NS: nonsignificant).

There are various pathways that control the internalization of nanoparticles by the tumor cells. They include caveolin-mediated endocytosis (Cav-ME), which is both dynamin- and cholesterol-dependent;^108^ clathrin-mediated endocytosis (CME); phagocytosis; and pinocytosis.^109^ In order to determine which of these mechanisms are involved in the internalization of L-aLPAR1, we treated 4T1 cells with three endocytosis inhibitors separately to block different specific endocytic pathways (Table 4) before treating with L-aLPAR1 and then measuring cellular uptake. Figure 3A shows that filipin III, a Cav-ME inhibitor, reduced the internalization of L-Ki16425 by 26% compared with the control. Meanwhile, dynasore (DNS), which inhibits both Cav-ME and CME, reduced the internalization of L-Ki16425 by 75%, compared with the control. Inhibiting phagocytosis and micropinocytosis by treatment with cytochalasin D (CCD) did not reduce the internalization of L-Ki16425. These results suggest that Cav-ME and CME are implicated in the internalization of L-Ki16425 by 4T1 cells but not phagocytosis and micropinocytosis.

**Table 4:** Endocytosis Inhibitors and Their Mechanisms of Action

inhibitor	action	mechanism
filipin III	inhibits Cav-ME	binds cholesterol^108^
DNS	inhibits CME and Cav-ME	blocks activity of GTPase in dynamin
CCD	inhibits phagocytosis and micropinocytosis	depolymerizes F-actin

### Tumor Accumulation of Liposome-Formulated LPAR1 Antagonists

Female BALB/c mice bearing MBC tumor masses were injected with one of the four liposomal formulations. The level of tumor accumulation in the liposomes was tracked for 48 h. Figure 3B,C shows that L-Ki16425 had the highest level of accumulation in tumor tissue compared with the other three formulations. At 6 h post-injection, L-Ki16425 exhibited 100% increase over L-DOPC. The same observation was true for all subsequent time points when tumor accumulation was measured until the end of the study. L-Ki16425 had a 130, 110, and 110% increase in tumor accumulation over L-DOPC at 9, 24, and 48 h, respectively.

At the end point of the study, the tumor masses and organs were dissected and imaged. Figure 3D shows that L-KI1645 had a 120% higher accumulation in tumor tissue, ex vivo, compared to L-DOPC, consistent with the data obtained in vivo (Figure 3B). The different liposomal formulations were then evaluated for their organ distribution. Figure 3E shows the distribution profile of liposomes in all of the organs represented as a percentage of the level of accumulation in the tumor tissue in each animal. While L-Ki16425 had increased tumor accumulation, no statistically significant differences were observed relative to the other formulations in the heart, lungs, liver, kidneys, stomach, spleen, brain, or intestine.

### MD Simulations of the Area Compressibility Modulus

We used MD simulations to investigate the structural and mechanical properties that may contribute to differences in cell–nanoparticle interactions among the different liposomal formulations. The Young’s modulus is often used to quantify the elasticity of lipid nanoparticles experimentally; it is derived from the initial slope of a stress versus strain curve. The area compressibility modulus (*K*_A_) measures the same property of the lipid bilayer in MD simulations. Herein, we investigated how the insertion of small drug molecules (i.e., AM095 and Ki16425) affected the elastic properties of the lipid membrane of liposomes. Drug–bilayer systems were simulated at 0, 5, 10, 15, and 20 mN/m surface tensions, where *K*_A_ is defined as the change in the surface tension per unit change in the area strain of the membrane.

**Figure 4:**
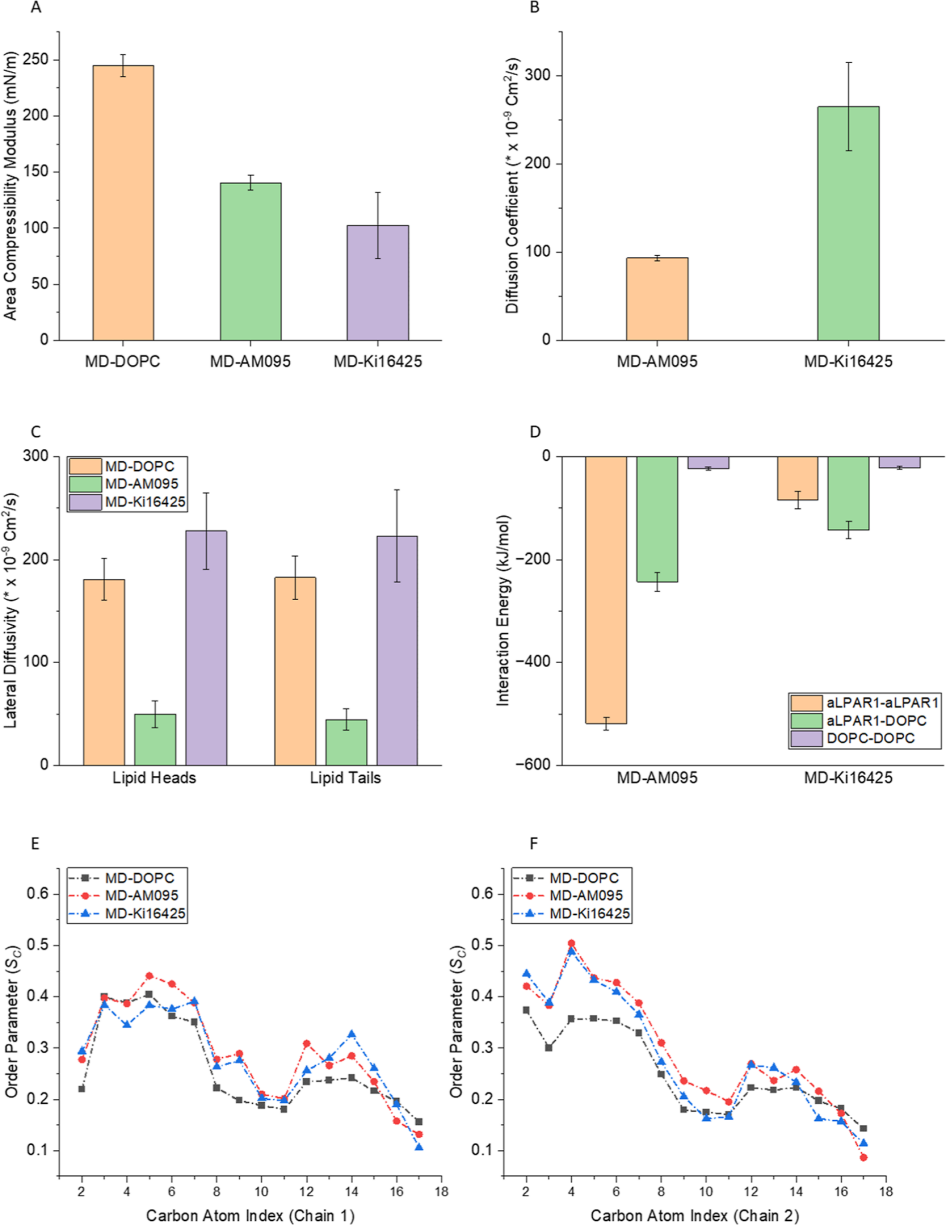
(A) Area compressibility moduli of MD-DOPC, MD-AM095, and MD-Ki16425. (B) Diffusion coefficients of AM095 and Ki16425 within the lipid bilayer. (C) Lateral diffusivity of the lipid bilayer for the pure DOPC system compared to those of the AM095 and Ki16425 systems. (D) Intermolecular interaction energy between drug molecules and lipids. (E) Order parameter of acyl chain 1 and (F) order parameter of acyl chain 2.

We calculated the *K*_A_ of the pure DOPC system (MD-DOPC) to be 245 ± 9.85 mN/m. This value is consistent with the results of previously reported simulation (256 ± 17 mN/m),^110^ as well as experimental data (265 ± 18 mN/m).^111^Figure 4A shows that the DOPC-AM095 (MD-AM095) and DOPC-Ki16425 (MD-Ki16425) systems have 43% and 58% lower *K*_A_, respectively, compared with the MD-DOPC system. Therefore, MD-DOPC demonstrated the highest stiffness among these systems. The presence of AM095 or Ki16425 resulted in a decrease in the stiffness of the lipid bilayer.

### MD Simulations of the Lateral Diffusion Coefficient and Interaction Energy

Diffusion coefficients can provide information about the mobility of molecules and thus the fluidity and ease of deformability of a given system. In this study, we treated the lipid bilayers as two-dimensional (2D) systems given the nanoscale thickness of the lipid membranes, in contrast to their larger lateral dimensions. Consequently, we examined the lateral diffusivities of AM095 and Ki16425 within the MD-AM095 and MD-Ki16425 systems, respectively. Figure 4B shows that the lateral diffusivity of the Ki16425 molecules within the lipid bilayer was approximately 3 times greater than that of the AM095 molecules. Furthermore, we calculated the lateral diffusion coefficients of both the headgroups and tails of the lipid molecules in the MD-DOPC, MD-AM095, and MD-Ki16425 systems to assess the effects of the addition of AM095 and Ki16425 on lipid fluidity.

The lateral diffusion coefficient (*D*_L_) of pure DOPC bilayers measured by nuclear magnetic resonance (NMR) was reported to be approximately 115 × 10^–9^ and 140 × 10^–9^ cm^2^/s at 303 and 333 K, respectively,^112^ while a value of 172 × 10^–9^ was reported using MD simulations.^113^ In this study, we measured the *D*_L_ of the pure DOPC bilayer to be 181 ± 20× 10^–9^ and 183 ± 21 × 10^–9^ cm^2^/s for the phosphorus atoms and acyl chains, respectively, which are in general agreement with reported experimental data considering that our chosen force-field parameters were not fitted to experimental diffusivities.^84,85^Figure 4C shows that the addition of AM095 or Ki16425 altered the lateral, 2D movement of the surrounding lipids, compared with the pure DOPC system. The addition of Ki16425 led to an approximately 20% increase in the movement of the phosphorus atoms (heads), as well as the acyl chains (tails) of the DOPC bilayer, whereas the incorporation of AM095 reduced their mobilities by approximately 3-fold. The higher *D*_L_ of DOPC in the MD-Ki16425 system suggests that the lipid molecules are highly mobile and diffuse rapidly, whereas the lower *D*_L_ in the MD-AM095 system suggests that the lipid molecules are tightly packed and have restricted mobility.

To investigate the origin of the observed differences in the diffusion coefficients between the drug and lipid molecules, we analyzed the interaction energies involved. Figure 4D shows that the AM095 molecules exhibited stronger intermolecular interactions among each other as well as with the lipid molecules, compared with Ki16425. These stronger interactions may hinder the mobilities of the AM095 and the lipid molecules in the MD-AM095 system compared with the MD-Ki16425 system.

### MD Simulations of Order Parameters

One of the most commonly used methods for studying the order in lipid membranes is the use of lipid chain order parameters. These order parameters offer insights into the orientation of the lipid chains relative to the bilayer normal. In this study, we examined the order parameter of acyl chain 1 and 2 of DOPC in the MD-DOPC, MD-AM095, and MD-Ki16425 systems, as shown in Figure 4E,F, respectively. The results show that the addition of AM095 and Ki16425 increased the order parameter of acyl chain 1 and 2, compared with pure DOPC, indicating that lipids were more vertically oriented, with a higher order parameter observed for acyl chain 2.

## Discussion

The LPA–LPAR axis has emerged as a promising target in cancer treatment. Given that the dysregulation of the expression of LPAR1 has been linked to multiple processes in the progression of various types of cancers, a drug delivery system that could target and deter this signaling pathway could prove useful in the treatment of MBC by hindering the proliferation and migration of cancer cells. Moreover, therapeutic tools that target specific cellular receptors on cancer cells may potentially be tailored to certain groups of patients in what is known as precision medicine.

In this study, we demonstrated that LPAR1 is overexpressed in 4T1 MBC cells compared with that in normal epithelial cells, EpH4-Ev (Figure 1A). We then investigated if and how the integration of LPAR1 antagonists (<500 Da) into the lipid bilayer of liposomes affected the cell–nanoparticle interactions with MBC cells. The data showed that L-Ki16425 had higher cellular internalization in mouse and human MBC cells in vitro (Figure 2A,B), as well as higher tumor accumulation in a murine syngeneic model for MBC (Figure 3B,C), compared with L-DOPC. Treatment of 4T1MBC cells with TNF-α increased L-Ki16425 internalization. TNF-α-treated 4T1 cells internalized L-Ki16425 90% more than L-DOPC, when incubated for 3 h (Figure 2C), while untreated 4T1 cells internalized L-Ki16425 48% more than L-DOPC, when incubated for 4.5 h (Figure 2A). Dysregulated cytokine production can increase cancer metastasis through a variety of mechanisms including angiogenesis, matrix remodeling, cell proliferation, and cell migration.^114^ TNF-α can alter membrane lipid composition^115^ and increase adhesion through both the upregulation of cell surface ligands^116−119^ and the activation of integrins by inside-out signaling.^119,120^ The increased uptake of L-Ki16425 in TNF-α-treated 4T1 cells may be attributed to the changes in cell membrane mechanics, increased adhesion, or changes in protein expression.

We subsequently focused our investigation on two main aspects that may contribute to the observed results, namely, the cellular mechanisms implicated in L-aLPAR1 internalization and the physical properties that may facilitate the interactions between L-aLPAR1 and cell membranes. First, we showed that the presence of LPA, the native LPAR1 ligand, hindered the uptake of L-Ki16425 in 4T1 cells (Figure 2D), which suggests that the uptake of L-Ki16425 is LPAR1-mediated. Inhibition experiments demonstrated that Cav-ME and CME were implicated in the internalization of L-Ki16425, while phagocytosis and micropinocytosis were not involved in the process (Figure 3A). To address the second aspect of our investigation, we used MD simulations to inspect the changes in the structural and mechanical properties associated with the integration of hydrophobic drug molecules into lipid bilayers. Our findings demonstrated that the integration of AM095 or Ki16425 within DOPC bilayers resulted in a decrease in compressibility modulus, more so in the case of Ki16425 (Figure 4A). MD data combined with the experimental results obtained in vitro and in vivo are in good agreement with our previous work where we showed that nanoparticle elasticity influenced cell–nanoparticle interactions, where soft nanoparticles (Young’s modulus < 1.6 MPa) showed higher cellular uptake and higher tumor accumulation compared with those of stiff nanoparticles (Young’s modulus > 13.8 MPa).^121^ Previous reports have shown that soft nanoparticles enhanced the circulation time and targeting in vivo compared to stiff nanoparticles.^122,123^

Our data showed that Ki16425 molecules had a greater diffusivity in the lipid bilayer compared to AM095 molecules (Figure 4B). Moreover, Ki16425 increased the lateral diffusion of lipids, while AM095 restricted their movement compared to the pure DOPC system (Figure 4C). Enhanced lipid movement is indicative of a more fluidic and, hence, softer bilayer. These observations indicate that AM095 may shift the lipid bilayer to a gel-like state, while Ki16425 may shift the lipid bilayer to a more fluid-like state. Both MD-Ki16425 and MD-AM095 had higher order parameter, especially for acyl chain 2, compared with MD-DOPC (Figure 4E,F). However, lipid packing is not the sole factor that influences the mechanical properties of a membrane as the data showed *K*_A_ to be lower for MD-AM095 and MD-Ki16425 compared with that for MD-DOPC. The integration of AM095 or Ki16425 within the lipid bilayer enhanced lipid packing, while at the same time, it disrupted the cohesive forces that hold the lipid molecules together. The addition of AM095 or Ki16425 reduced the average interaction energy per molecule between lipids from 25.12 ± 5.33 to −22.33 ± 2.82 and −21.03 ± 3.26 kJ/mol per lipid, respectively. In addition, the interactions between the molecules of each antagonist within the lipid bilayer were significantly different, having an average interaction energy per molecule of −518.81 ± 12.66 and −84.49 ± 17.05 kJ/mol for AM095 and Ki16425, respectively. The differences in the physical and mechanical properties between L-Ki16425, L-AM095, and L-DOPC as evidenced by the data of the MD simulations may help explain the disparities between their respective levels of cellular internalization (Figure 2A,B) and tumor accumulation (Figure 3B,C).

Overall, these results offer valuable insights into the molecular behavior of the drug–lipid bilayer complexes and highlight some of the factors that may contribute to the way they interact with living systems.

## Conclusions

In this study, we showed that the integration of the small molecules (<500 Da) of an LPAR1 antagonist, Ki16425, into the lipid bilayer of liposomal vesicles enhanced liposomal uptake by MBC cells in vitro and tumor accumulation in a mouse model of MBC. MD simulations showed that the addition of Ki16425 molecules made the liposomal vesicles softer and more deformable. This work lends insights into how to engineer more efficient drug delivery systems that may aid in the treatment of MBC.

## Supporting Information Available

Radial distribution function of 10, 15, 20, 30, and 40 molecules of AM095 and Ki16425 in DMSO; nomenclature of atoms in AM095 and Ki16425; snapshots of the simulations of the DOPC-AM095 and DOPC-Ki16425 systems; and snapshots of the simulations of 10, 15, 20, 30, and 40 molecules of AM095 and Ki16425 in DMSO (PDF)
