# Supplementary material for: Integration of an LPAR1 Antagonist into Liposomes Enhances Their Internalization and Tumor Accumulation in an Animal Model of Human Metastatic Breast Cancer
Source: Mol Pharm. 2023 Oct 16;20(11):5500–14. doi: 10.1021/acs.molpharmaceut.3c00348 (PMC10631474; doi:10.1021/acs.molpharmaceut.3c00348)
Supplement: Supplementary file 1 — mp3c00348_si_001.pdf [file mp3c00348_si_001.pdf]

Supporting Information for the Manuscript Entitled:

Integration of an LPAR1 Antagonist into Liposomes Enhances Their Internalization and Tumor Accumulation in an Animal Model of Human Metastatic Breast Cancer

Rudolf G. Abdelmessih[a], Jiaming Xu[a], Francisco R. Hung[a] and Debra T. Auguste[a*]

[a]Department of Chemical Engineering, Northeastern University, 360 Huntington Ave, Boston, Massachusetts 02115, United States

[*]Email: d.auguste@northeastern.edu

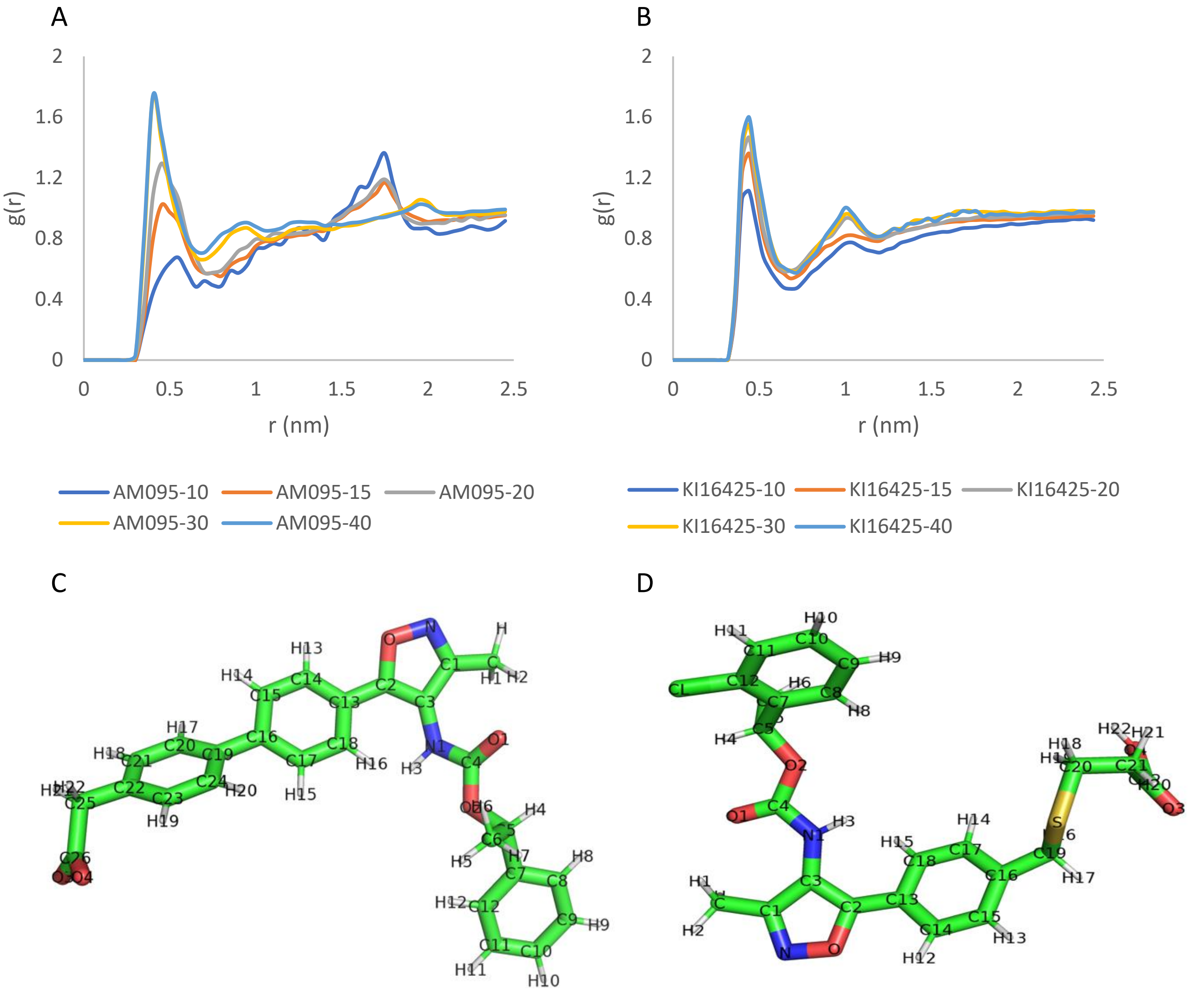

Figure S1. Radial distribution function of 10, 15, 20, 30 and 40 molecules of **(A)** AM095 and **(B)** Ki16425 in DMSO, and nomenclature of atoms in **(C)** AM095 and **(D)** Ki16425. The atom labeled as 'C' was utilized in the calculation of the radial distribution function for the results displayed in both **(A)** and **(B)**

A B

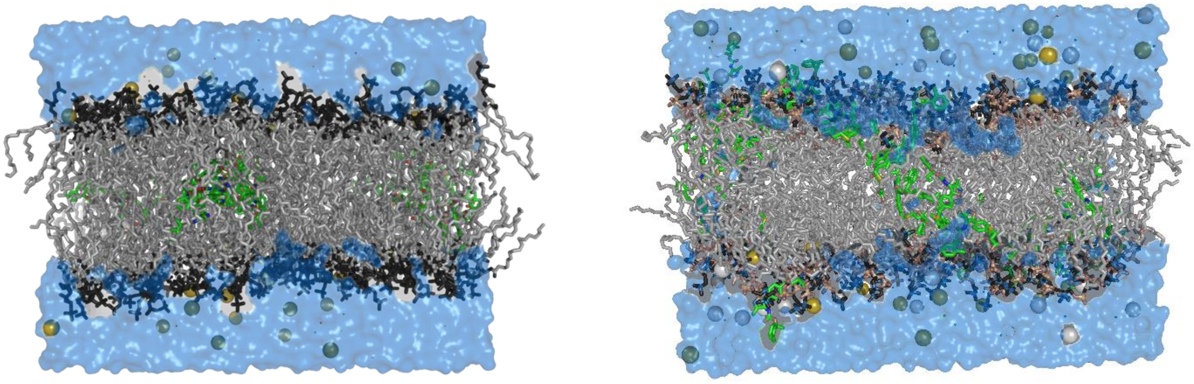

Figure S2. **(A)** A snapshot of the simulation of the DOPC-AM095 system. **(B)** A snapshot of the simulation of the DOPC-AM095 system. Hydrogen atoms omitted for clarity. In the images, the solvents are represented as transparent blue surfaces while the drug molecules are colored according to their constituent elements: carbon atoms are green, nitrogen atoms are blue, phosphorous atoms are yellow, and oxygen atoms are red. The lipid tails are depicted in gray, while the phosphorus groups and headgroups are shown in black. Sodium and chloride ions are depicted as small and large unbonded particles in gold.

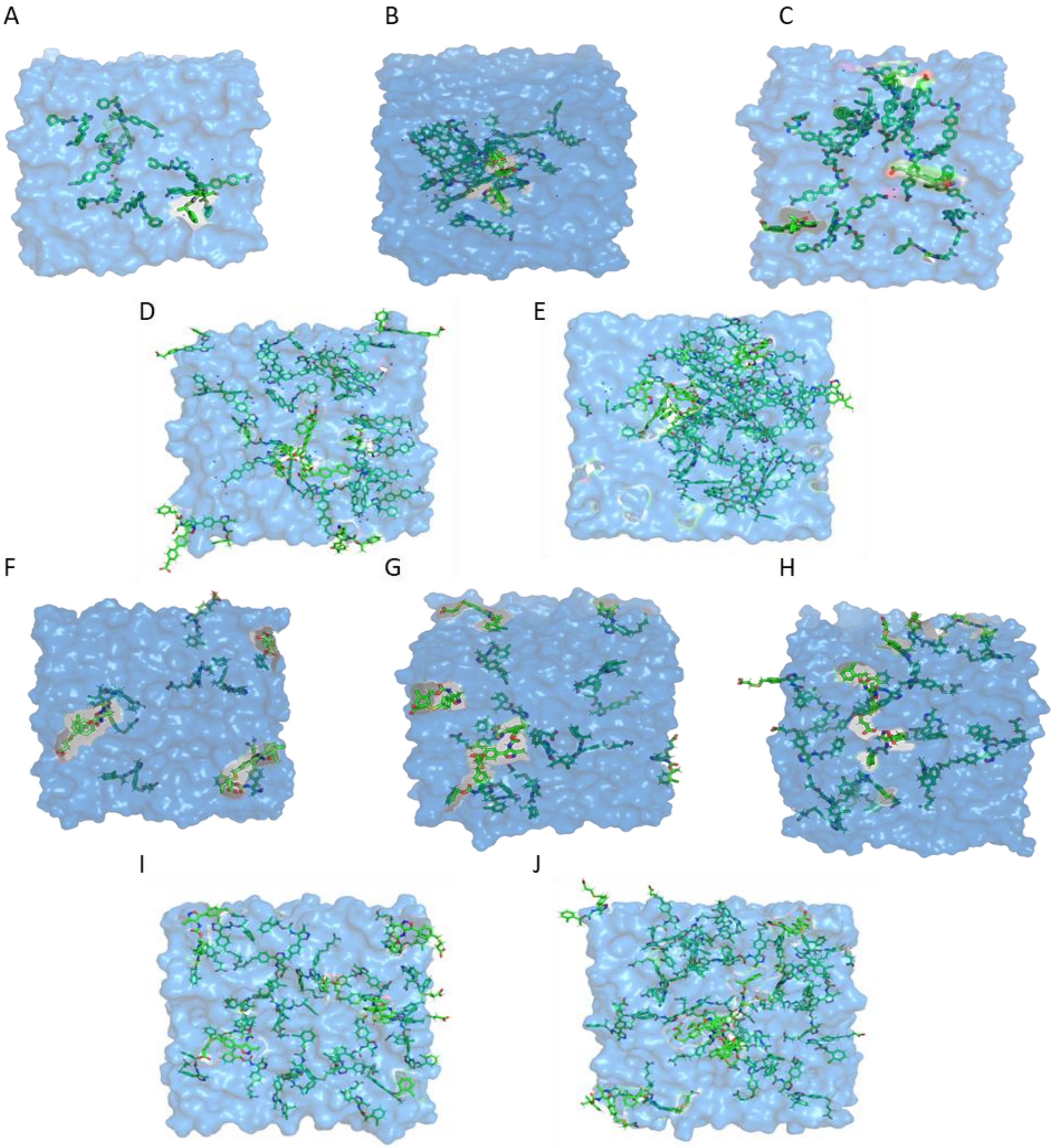

Figure S3. **(A) – (E)** show snapshots of the simulation of 10, 15, 20, 30 and 40 molecules of AM095 in DMSO, respectively. **(F) – (J)** show snapshots of the simulation of 10, 15, 20, 30 and 40 molecules of Ki16425 in DMSO, respectively. Hydrogen atoms omitted for clarity. In the images, the solvents are represented as transparent blue surfaces while the drug molecules are colored according to their constituent elements: carbon atoms are green, nitrogen atoms are blue, phosphorous atoms are yellow, and oxygen atoms are red. The lipid tails are depicted in gray, while the phosphorus groups and headgroups are shown in black. Sodium and chloride ions are depicted as small and large unbonded particles in gold.

References

1. Project, T.G.o.D.S.i.C. *Genomics of Drug Sensitivity in Cancer_Compound: Cisplatin*. [cited 2023 January 23]; Available from: https://www.cancerrxgene.org/compound/Cisplatin/1005/overview/ic50.
2. Project, T.G.o.D.S.i.C. *Genomics of Drug Sensitivity in Cancer_Compound: Paclitaxel*. [cited 2023 January 23]; Available from: https://www.cancerrxgene.org/compound/Paclitaxel/1080/overview/ic50.
3. Project, T.G.o.D.S.i.C. *Genomics of Drug Sensitivity in Cancer_Compound: Doxorubicin*. [cited 2023 January 23]; Available from: https://www.cancerrxgene.org/compound/Doxorubicin/133/overview/ic50.
4. Furumai, R., et al., *FK228 (depsipeptide) as a natural prodrug that inhibits class I histone deacetylases.* Cancer Res, 2002. **62**(17): p. 4916-21.
5. Sun, W.J., et al., *Romidepsin induces G2/M phase arrest via Erk/cdc25C/cdc2/cyclinB pathway and apoptosis induction through JNK/c-Jun/caspase3 pathway in hepatocellular carcinoma cells.* Biochem Pharmacol, 2017. **127**: p. 90-100.
6. Crisanti, M.C., et al., *The HDAC inhibitor panobinostat (LBH589) inhibits mesothelioma and lung cancer cells in vitro and in vivo with particular efficacy for small cell lung cancer.* Mol Cancer Ther, 2009. **8**(8): p. 2221-31.
